# Clinical characteristics and temporal trends of meropenem and piperacillin/tazobactam heteroresistance in *Pseudomonas aeruginosa* isolates

**DOI:** 10.1186/s12941-026-00875-2

**Published:** 2026-06-26

**Authors:** Yali Fan, Huilan Li, Xiaoyue Liang, Jia Xie, Biyu Lin, Zheng Liang, Weifeng Liu, QinJin Wang, Tian Zhong, Ying Chen, Shuping Nie, Chao Wu

**Affiliations:** 1 Department of Laboratory Medicine, the Eighth Affiliated Hospital, Sun Yat-sen University, Shenzhen, China; 2 Biological Laboratory of Hetao Cooperation Zone, the Eighth Affiliated Hospital of Sun Yat-sen University, Shenzhen, China

**Keywords:** Heteroresistance, *Pseudomonas aeruginosa*, Epidemiological characteristics, Phenotypic characteristics, Clinical factors, Antimicrobial stewardship

## Abstract

**Background:**

Heteroresistance (HR) to commonly used anti-pseudomonal agents in *Pseudomonas aeruginosa* has been reported in cross-sectional epidemiological surveys; however, longitudinal studies investigating the long-term epidemiological dynamics and clinical correlates of this adaptive phenotype are still lacking. This retrospective time-series study aims to systematically explore the HR phenotypic profiles and temporal dynamic of clinical *P. aeruginosa* isolates against piperacillin/tazobactam (TZP) and meropenem (MEM), and further clarify their potential clinical correlates.

**Methods:**

We comprehensively analyzed 380 non-duplicate clinical strains collected from a tertiary hospital between 2011 and 2024. The disk diffusion and Etest gradient diffusion methods were used for the preliminary determination of HR incidence rates in clinical isolates against six anti-pseudomonal antibiotics. Population analysis profiling (PAP) assays was further performed to assess the temporal dynamics and phenotypic characteristics of HR to TZP MEM. HR was defined as the presence of resistant subpopulations with an MIC at least eightfold higher than that of the dominant population at a frequency of ≥ 1 × 10⁻⁷. Subsequently, univariate and multivariate regression analyses were performed to identify clinical factors associated with HR and non-HR strains.

**Results:**

Preliminary screening indicated that 55% of the tested strains exhibited the HR phenotype, whereas confirmatory assays further verified that 49.5% of the isolates displayed HR to either TZP or MEM. The HR detection rate for MEM peaked at 35.3% during 2017–2019, followed by a gradual decline, reaching a minimum of 24.04% during the 2020–2022 pandemic. Meanwhile, the HR rate to TZP initially increased, then decreased, peaking at 43.1% during 2017–2019 and subsequently declining to 27.8%. In addition, multivariate regression analysis identified that male gender and prior antiviral exposure were independently associated with decreased risk of HR, whereas prior fluoroquinolone antibiotics exposure was independently associated with an increased risk.

**Conclusion:**

Our findings highlight the widespread prevalence, distinct temporal dynamics, and diverse phenotypic features of HR, and underscore the potential clinical value of integrating HR screening into antimicrobial susceptibility testing, particularly for high-risk patient populations, to better guide therapy and mitigate the risk of treatment failure.

**Supplementary Information:**

The online version contains supplementary material available at https://doi.org/10.1186/s12941-026-00875-2.

## Background

*Pseudomonas aeruginosa* is a significant nosocomial pathogen characterized by intrinsic and acquired resistance to multiple antimicrobial agents, which poses substantial challenges to antimicrobial therapy in healthcare settings [1, 2]. In addition, previous studies have demonstrated that *P. aeruginosa* can generate small subpopulations (less than 0.1% of the total population) with increased antibiotic resistance under continuous antibiotic exposure, potentially leading to treatment failure [3]. This phenomenon, termed heteroresistance (HR), was defined in this study as the presence of resistant subpopulations exhibiting at least an eightfold higher antibiotic MIC than the dominant population at a frequency of ≥ 1 × 10⁻⁷. HR presents substantial detection challenges because conventional susceptibility testing frequently fails to identify low-abundance resistant subpopulations [4, 5]. Consequently, this diagnostic gap risks misclassifying heteroresistant strains as susceptible, potentially triggering inappropriate antibiotic use and subsequent treatment failure [6–8]. Although HR was first identified in 1947—nearly 75 years ago—it remains severely underestimated and inadequately recognized in clinical microbiological practice, primarily due to its ambiguous definition and inherent detection challenges [9].

HR has been detected in a range of clinical bacterial strains, including *Staphylococcus aureus* [10], *Acinetobacter baumannii* [11], *Klebsiella pneumoniae* [12], *Escherichia coli* [13], and *P. aeruginosa* [14, 15], and these organisms often exhibit HR to multiple antibiotics. Previous studies have demonstrated that *P. aeruginosa* exhibited HR to β-lactams, carbapenems, fluoroquinolones, and colistin. He J reported an exceptionally high HR rate to meropenem (MEM) in invasive *P. aeruginosa* strains (72.5%), with a significant increase in the prevalence of MEM-HR isolates during 2011–2015. Additionally, carbapenem consumption showed a dose-dependent association with carbapenem HR prevalence in *P. aeruginosa* [14]. HR to piperacillin-tazobactam (TZP) has been reported in *Klebsiella pneumoniae* and *Escherichia coli* [13, 16]. However, comprehensive studies on TZP-HR in *P. aeruginosa* remain lacking. Although accumulating evidence indicates that HR prevalence fluctuates with antibiotic usage and over time, no studies has examined the long-term temporal trends of HR in *P. aeruginosa* spanning more than a decade, particularly in China. In addition, limited data exist on the epidemiological characteristics of HR in clinical *P. aeruginosa* isolates, which are critical for elucidating the distribution and burden of HR in clinical practice. Furthermore, clinically relevant factors associated with *P. aeruginosa* HR infections remain incompletely characterized.

Given the limited number of studies examining the temporal trends of HR in *P. aeruginosa*, we conducted the first longitudinal analysis to elucidate the time dynamics of HR incidence using a long-term cohort. This study analyzes 14 years of clinical isolates to systematically assess the prevalence and phenotypic characteristics of HR profiles, with a dedicated focus on MEM and TZP. We further evaluated the temporal evolution of HR rates and their correlations with clinical factors. These findings are anticipated to provide a basis for the rational use of antibiotics in clinical practice, facilitate prudent antimicrobial utilization, and improve treatment outcomes. Furthermore, this work is expected to offer novel insights into the temporal dynamics of HR in *P. aeruginosa* and provide new directions for investigating the underlying mechanisms of HR.

## Materials and methods

### Setting and study design

**Fig. 1:**
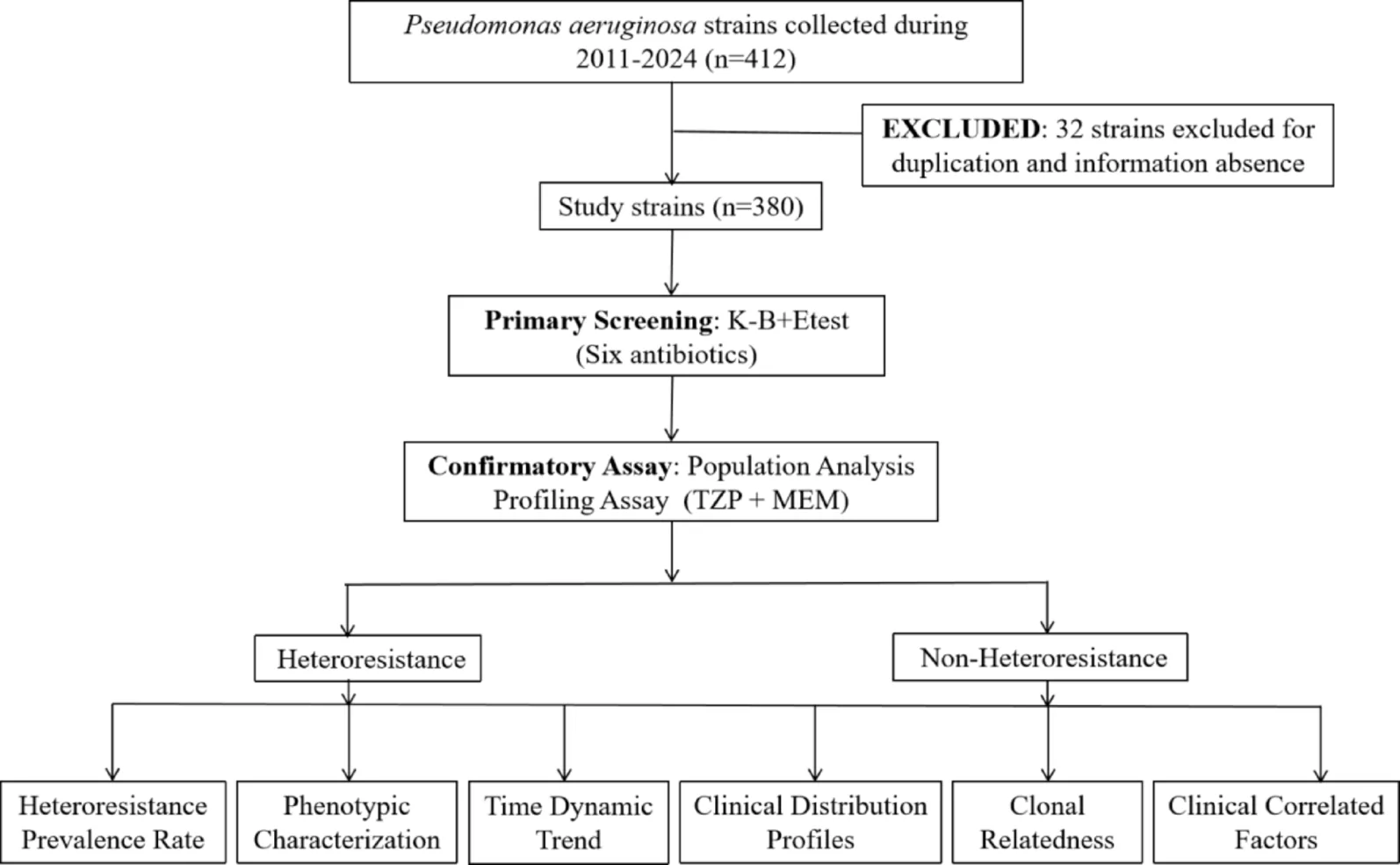
Overview of study design

This retrospective study of HR in *P. aeruginosa* strains was conducted from May 2011 to June 2024 at a tertiary hospital in Shenzhen. All *P. aeruginosa* strains were collected from the Clinical Laboratory Medicine Department. Then excluded duplicate isolates from the same patient, only retaining the first strain, and patients with incomplete clinical data. A total of 380 non-duplicate *P. aeruginosa* strains were identified for subsequent HR analysis. Furthermore, preliminary screening for HR was performed against six commonly used anti-Pseudomonas antibiotics, followed by specific validation of TZP and MEM using the population analysis profile (PAP) assay. Representative strains were further selected for cloning correlation analysis. Eligible demographic date, clinical diagnosis and antimicrobial therapy were collected to elucidate the clinical correlated factors of HR in *P. aeruginosa* (Fig. 1). This study was approved by the Medical Ethics Committee of the Eighth Affiliated Hospital of Sun Yat-sen University.

### Preliminary screening of heteroresistance

All isolates were identified using the VITEK-2 compact automated system (bioMerieux) in clinical laboratory testing. To preliminarily assess HR to six anti-Pseudomonas antibiotics, the Kirby-Bauer disk diffusion method (K-B, Oxoid) and the Etest gradient diffusion assay (Etest, Biomérieux) were employed. To preliminary isolate potential HR strains, 0.5 McFarland standard bacterial suspensions were inoculated onto Mueller–Hinton agar (MHA; Jiangmen Bio-Caring), followed by placement of six antibiotic-impregnated paper disks. After incubation at 37 °C for 18 h, distinct colonies growing within the inhibition zone were selected. The minimum inhibitory concentrations (MICs) of the resistant subpopulation (zone-internal colonies) and the parent population (zone-external colonies) were re-assessed using Etest. Strains exhibiting ≥ 4-fold MIC difference between the two populations were classified as potential HR, otherwise, classified as NHR [4]. The *P. aeruginosa* reference strain ATCC 27,853 was utilized as a control.

### Population analysis profiling

The gold standard PAP method was employed to detect HR of *P. aeruginosa* to TZP and MEM, according to a previously published protocol with several modifications [4, 17]. To minimize false-negative results from preliminary screening, PAP was performed on all 380 isolates included in this study. Briefly, the analysis was performed by spreading approximately 10^8^ CFUs on MHA plates with TZP in serial dilutions at concentrations ranging from 2/4 mg/L to 256/4 mg/L, with MEM at concentrations from 0.25 mg/L to 32/4 mg/L. We rigorously defined HR according to the presence of subpopulations capable of growing at concentrations of antibiotics at least eightfold higher than the highest concentration that does not affect the replication of the dominant population, with the frequency of resistant subpopulations is 1 × 10^− 7^ or higher. The analysis was performed for these selected isolates in three replicates, and ATCC 27,853 strain was used as the control.

### Analysis of clonal relatedness

To assess clonal relatedness between the parent populations and resistant subpopulations of TZP-HR and MEM-HR *P. aeruginosa* isolates based on genes sequence similarity, six common resistant genes of *P. aeruginosa* were selected, including AmpC, mexB (MexAB-oprM), oprD, ftsL, gyrA and Aph. The phylogenetic tree was generated using sequence alignment data from the six resistance genes, which were aligned using ClustalW. The resulting alignment was subjected to phylogenetic analysis using Maximum Likelihood method, with branch lengths reflecting the genetic divergence among the strains. The PCR amplification primers and reaction conditions used are shown in Table S1.

### Data collection and analysis variables

Clinical and demographic data were retrospectively extracted from the electronic medical records system. Employing univariate regression analysis to evaluate the clinical characteristics of HR and NHR patients with *P. aeruginosa* infection. Cases and controls were compared regarding the following parameters: demographics; comorbidities and underlying diseases; invasive procedures within the prior 4 weeks and prior antibiotic exposure within 1 months (before the first positive culture was obtained). Spearman rank correlation analysis was performed to evaluate the associations between population level HR rates and clinical outcome indicators (intensive care unit (ICU) admission rate, treatment failure rate, mortality rate, median total length of hospital stay and median postculture length of stay) across 5 distinct annual time points. Given the limited number of aggregate time-period observations (*n* = 5), the results of this analysis are presented as exploratory temporal trends rather than definitive causal associations.

### Statistical analysis

Univariate logistic regression analysis was conducted for all variables. Categorical variables were coded as 1 (“positive/present”) and 0 (“negative/absent”), with the “negative/absent” category serving as the reference group. For categorical variables, the Chi-square (χ^2^) test was utilized to assess differences, with results expressed in terms of counts and proportion. Continuous variables were compared using Student’s t-test (normally distributed variables) and the Wilcoxon rank-sum test (non-normally distributed variables) and were presented as median values (M), along with first (Q1) and third (Q3) quartiles. Univariate analysis selected variables with *p* < 0.1, as potential covariates in a stepwise multivariate logistic regression model. Odds ratios (ORs) and 95% confidence intervals (CIs) were calculated for each factor. Spearman’s correlation coefficient (ρ) was calculated to quantify the strength and direction of the correlation. All statistical analyses were conducted with IBM SPSS Statistics 20.0 (IBM Corporation, Armonk, NY, USA), with a two - tailed *P* value < 0.05 considered statistically significant.

## Results

### The prevalence of HR in clinical *P. aeruginosa* strains

A total of 380 clinically isolated *P. aeruginosa* strains collected from 2011 to 2024 were included in this study. Our preliminary screening results indicated that HR is a common phenomenon among *P. aeruginosa* isolates based on the K-B and Etest assays, with significant variation in HR detection rates observed across different antibiotics. Initially, 73.42% of the 380 clinical strains were identified as HR using the K-B method. However, after confirmation with a combined K-B and Etest approach, the proportion of HR among these isolates was 55.0% (Table 1). Among the tested antibiotics, the detection rate of HR to MEM was the highest at 25.26%, followed by TZP (23.68%) and CAZ (17.11%). The detection rate of HR by the K-B method was significantly higher than that obtained with the combined K-B and Etest assays (73.42% VS 55.00%, *P* < 0.001), with an overall concordance rate of 75% between the two methods.

**Table 1:** Preliminary screening: heteroresistant rates of *P.aeruginosa* strains against different antibiotics

Methods	Total HR	MEM HR	TZP HR	FEP HR	CAZ HR	LEV HR	AK HR
K-B	279/380(73.42%)	159(41.84%)	143(37.63%)	111(29.21%)	76(20%)	63(16.58%)	65(17.11%)
K-B+ Etest	209/380(55.00%)	96(25.26%)	90(23.68%)	49(12.89%)	65(17.11%)	51(13.42%)	16(4.21%)

K-B, Kirby-Bauer disk diffusion method; Etest: Etest gradient diffusion assay; MEM, meropenem; TZP, piperacillin/tazobactam; FEP, cefepime; CAZ, ceftazidime; LEV, levofloxacin; AK, amikacin

**Fig. 2:**
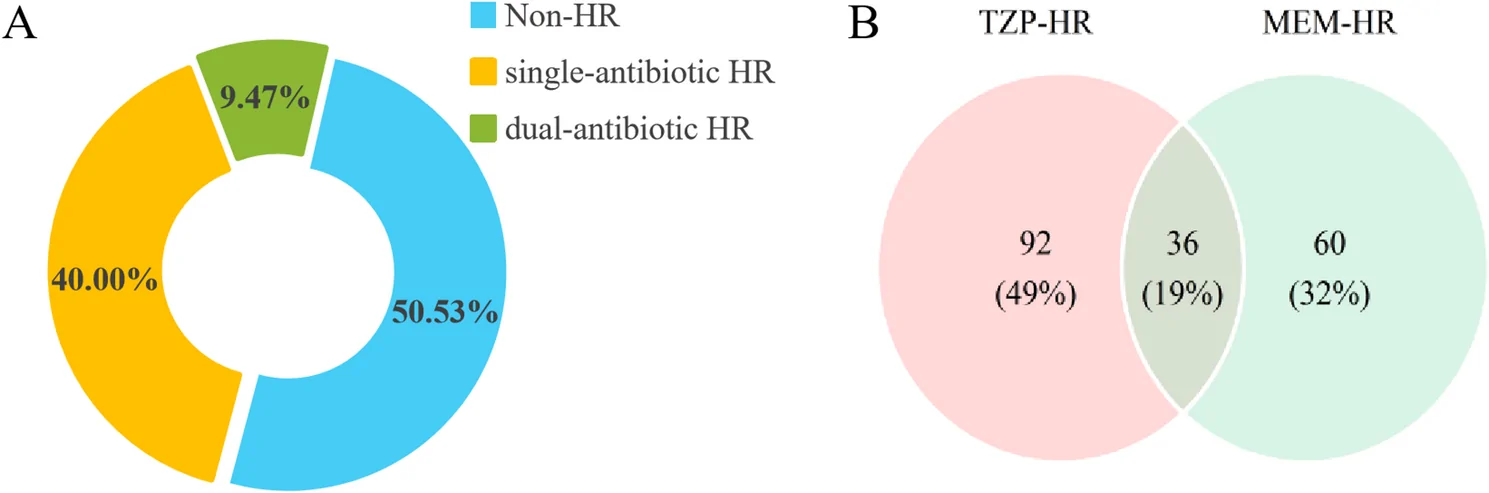
Verification of heteroresistance to TZP and MEM in clinical *P. aeruginosa* isolates. **A**. Proportion of *P. aeruginosa* strains with heteroresistance to TZP and MEM. **B**. Quantification of single (TZP or MEM) and dual-antibiotic heteroresistant strains

To further validate the HR phenotype, the PAP assay - the gold standard method was performed to assess the prevalence of HR to TZP and MEM in *P. aeruginosa* isolates (Fig. S1). The PAP assay for TZP showed that heteroresistant isolates were able to grow at concentrations ranging from 64 to 256 mg/L (with tazobactam fixed at 4 mg/L), whereas MEM-HR subpopulations proliferated at MEM concentrations from 8 to 32 mg/L. The results of the confirmatory assays demonstrated that 49.47% of the tested strains exhibited an HR phenotype, with 9.47% of these isolates displaying dual-antibiotic HR simultaneously (Fig. 2). Among all HR strains, 68% (128/188) exhibited HR to TZP, 51% (96/188) displayed MEM-HR, and 19% (36/188) further showed cross-heteroresistance (dual-antibiotic HR phenotype) to both antibiotic agents. This finding underscores the substantial overlap in resistant patterns between the two antibiotics, as well as the high likelihood of cross-reactivity in HR profiles.

### In vitro phenotypic characterization of heteroresistant populations

**Fig. 3:**
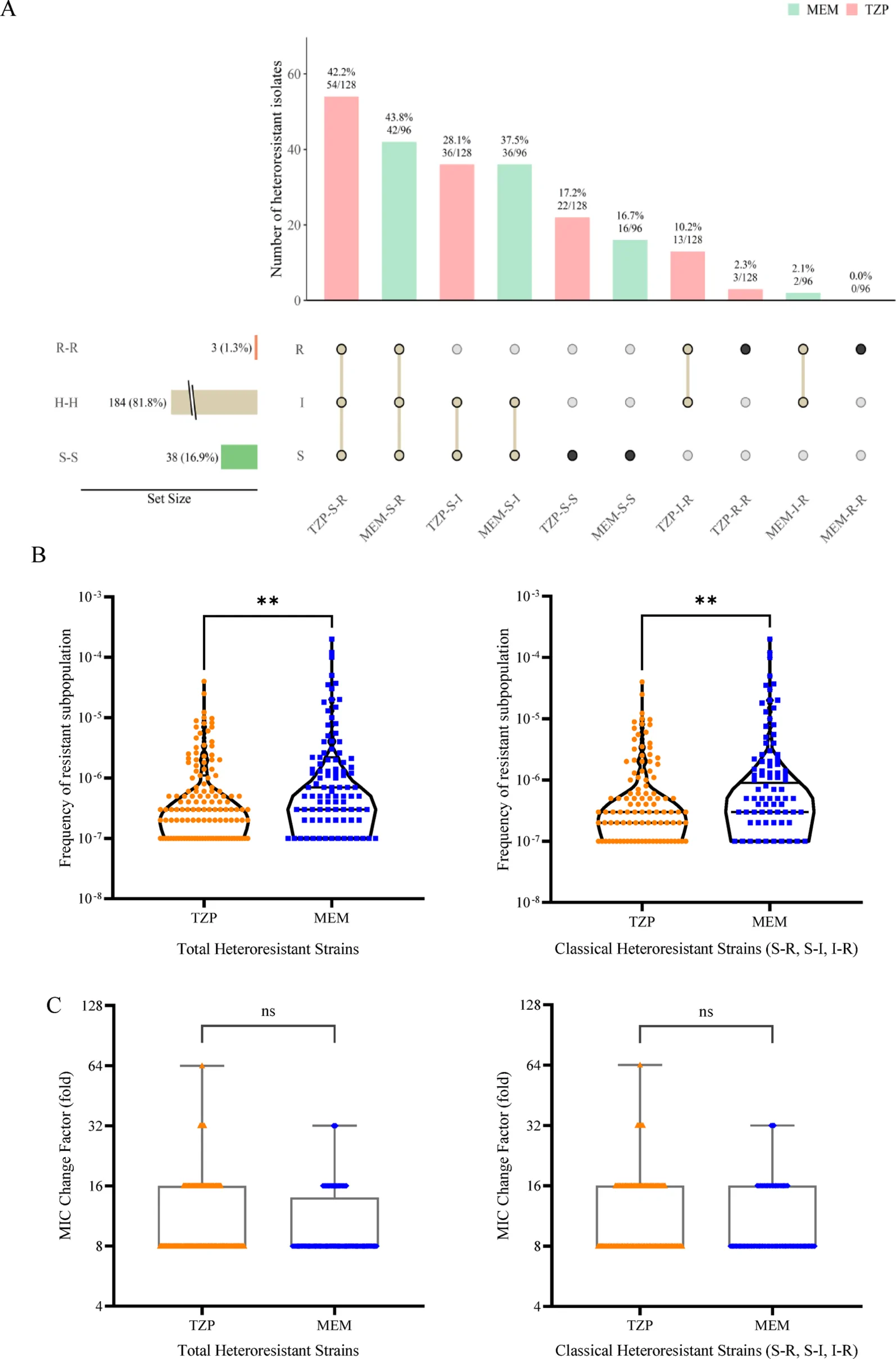
In vitro phenotypic characterization of the heteroresistant subpopulations. **A**. Changes in antibiotic susceptibility and quantitative distribution profiles of *P. aeruginosa* heteroresistant subpopulations to TZP and MEM. The Lower panel depicts the five types of antibiotic phenotypic changes observed in the HR subgroups. For example,“S-R” denotes that, for HR strains, the predominant population exhibits a susceptible phenotype, whereas the resistant subpopulation displays a resistant phenotype. Profiles include susceptible to reduced susceptibility (S-S), susceptible to intermediate susceptibility (S-I), intermediate to resistant transition (I-R), susceptible to resistant (S-R), and resistance to higher-level resistance (R-R). The upper bar chart shows the number of HR isolates corresponding to each subgroup (MEM, green bars; TZP, pink bars). Auxiliary metrics are also annotated to supplement phenotypic features. The H-H group included strains with heterogeneous resistance phenotypes (S-I, S-R, I-R), which were distinct from the homogeneous phenotypes of S-S and R-R groups. Numbers above each bar indicate the percentage of each phenotype within the group, along with the corresponding absolute counts, presented as “number of HR-positive isolates / total HR isolates” for each phenotype. **B** Frequency distribution of heteroresistant subpopulations of total heteroresistant strains (left) and classical heteroresistant strains (right). Violin plots show the frequency of resistant subpopulations for each strain: orange for TZP group and blue for MEM group. The y-axis is on a log10 scale, representing the frequency of resistant subpopulations. Mann–Whitney U test: **P < 0.01. **C**. Fold change in MIC of heteroresistant subpopulations of total heteroresistant strains (left) and classical heteroresistant strains (right). ns: no significant

Changes in antibiotic susceptibility and quantitative distribution profiles of *P. aeruginosa* heteroresistant subpopulations to TZP and MEM were shown in Fig. 3A. The lower panel illustrated the five phenotypic profiles of subpopulations within HR isolates, while the upper bar chart displayed the number of HR isolates per subgroup. The results showed that the S-R (susceptible to resistant) profile was most prevalent pattern in both subsets: 42.2% (54/128) of TZP-HR and 43.8% (42/96) of MEM-HR isolates fell into this profile. The S-I (susceptible to intermediate) profile was second, accounting for 28.1% of TZP-HR and a slightly higher 37.5% of MEM-HR isolates. Proportions of isolates with the S-S (susceptible to reduced susceptibility) phenotype were similar between groups (17.2% vs. 16.7%). Finally, the R-R (resistance to higher-level resistance) phenotype was restricted to TZP-HR isolates (3/128, 3.82%) and absent among MEM-HR isolates. Collectively, increased resistance among HR subpopulations (S-R, S-I, and I-R profiles) predominated the susceptibility change patterns in both TZP-HR and MEM-HR (approximately 81%), with modest differences in profile distribution between the two antibiotic-specific HR subsets. Notably, these resistant subpopulations may act as key drivers of eventual treatment failure. The violin plots illustrated the frequency of resistant subpopulations in HR strains to TZP and MEM, with each data point representing an individual HR strain (Fig. 3B). For TZP-HR strains, the proportion of resistant subpopulations that were able to grow at the highest tested antibiotic concentrations ranged from 10^− 7^ to 4 × 10^− 5^, whereas the maximum frequency of MEM resistant subpopulations reached 2 × 10^− 4^. Meanwhile, the frequency distribution range of resistant subpopulations was consistent between classical HR strains and the total HR strains. A statistically significant difference was observed in the frequency distribution of resistant subpopulations between the TZP and MEM groups (*P* < 0.01), suggesting that MEM selection pressure was associated with greater heterogeneity in the abundance of resistant subpopulation within HR strains. The fold change in MIC of heteroresistant subpopulations among total heteroresistant strains (left) and classical heteroresistant strains (right) were shown in Fig. 3C. In TZP heteroresistant strains, 65.6% of resistant subpopulations exhibited an MIC that was 8-fold higher than that of the parental strain, 29.7% showed a 16-fold increase, and fewer than 5% demonstrated an MIC elevation of ≥ 32-fold relative to the parental strain. A comparable pattern was observed among MEM heteroresistant strains, in which 75.0% of resistant subpopulations exhibited an 8-fold increase in MIC compared with the parental strain, 21.9% showed a 16-fold increase, and 3.1% reached a 32-fold elevation in MIC. A similar distribution pattern was observed among classical heteroresistant strains: 94.3% of resistant subpopulations in TZP-HR strains exhibited MICs that were 8-16-fold higher than that of the parental strains, while this proportion reached 97.2% in MEM-HR strains, with no statistically significant difference between the two groups.

### Trends in the detection rate of heteroresistance in *P. aeruginosa* strains

**Fig. 4:**
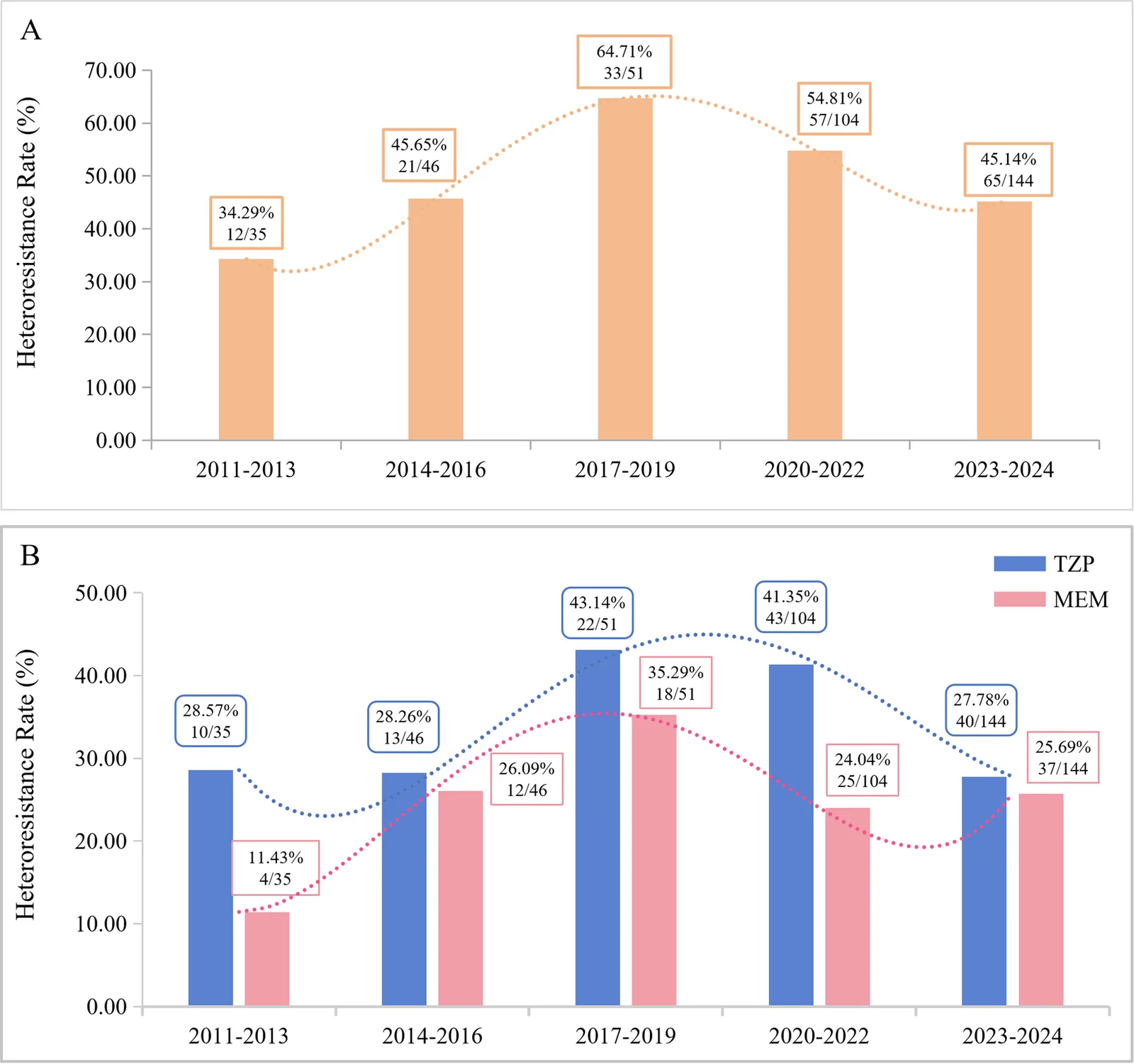
Temporal trends in the detection rates of HR from 2011 to 2024. Numbers above each bar indicate the heteroresistance rate and the corresponding counts of HR-positive isolates/total isolates for each time period. **A** .Overall detection rates and temporal trends of HR to two anti-pseudomonal agents. The bar chart represented the detection rate of HR, while the trend line illustrated the temporal trends in the HR detection rate over time, with a time interval of three years. **B**. Detection rates and temporal trends of MEM and TZP heteroresistance in clinical *P. aeruginosa* isolates. Blue: TZP heteroresistance; Pink: MEM heteroresistance

We further analyzed the temporal dynamic trends of HR rates to TZP and MEM in *P. aeruginosa* strains by dividing the 2011–2024 period into five consecutive three-year intervals, a segmentation that incorporates data from the 2020–2022 COVID-19 pandemic period. The overall detection rate of HR remained relatively high, exhibiting a dynamic trend of initial increase followed by a decrease, and reached a peak value of 64.71% during 2017–2019 (Fig. 4A). Our results indicate that HR rates not only fluctuated significantly over time but also showed significant differences across different antibiotics.

The temporal trend of the TZP-HR rate exhibited distinct fluctuating characteristics across the study intervals. During 2011–2013, the baseline TZP-HR rate was 28.57%. It then followed a continuous upward trend, peaking at 43.14% in 2017–2019. Subsequently, it gradually declined, eventually dropping to 27.78%—a level approaching the baseline documented in 2011–2013. TZP is a preferred empiric therapeutic agent for *P. aeruginosa* infections owing to its broad antibacterial spectrum and excellent activity against this pathogen. Its widespread clinical application may be related to the persistently high HR rate to TZP.

The detection rate of MEM-HR exhibited more pronounced fluctuations over the entire study period, with the overall HR rate consistently lower than that of TZP across all time intervals. Starting from a low baseline of 11.43% in 2011–2013, it increased sharply to a peak of 35.29% in 2017–2019, then subsequently declined to 24.04% during the pandemic period. This reduction may be associated with the implementation of stringent infection prevention and control measures and prudent use of antibiotics during the pandemic. Collectively, these results emphasized the dynamic and antibiotic-specific characteristics of HR in clinical *P. aeruginosa* strains, highlighting the spatiotemporal heterogeneity of HR profiles and underscoring the need for antibiotic-specific surveillance and management strategies in clinical practice.

### Clinical distribution profiles of heteroresistance in *P.aeruginosa* strains

The demographic (age and gender) distribution profiles of heteroresistant *P. aeruginosa* strains were documented in Table S2. Among all age groups, the ≤ 60-year cohort exhibited the highest HR detection rate, reaching 54.46% (61/112), whereas the lowest rate was found in the ≥ 80-year group, at 44.44% (48/108). Despite this apparent numerical variation, no statistically significant difference in HR detection rates was detected among the study groups. Approximately two-thirds of the enrolled patients were male, accounting for 68.95% of the total cohort (262/380). Intriguingly, the HR detection rate was significantly higher in females than in males (58.47% VS 45.42%, P<0.05). However, HR was detected in all demographic subgroups evaluated, indicating that heteroresistance is widely distributed among clinical *P. aeruginosa* isolates rather than being restricted to specific patient populations. 

**Fig. 5:**
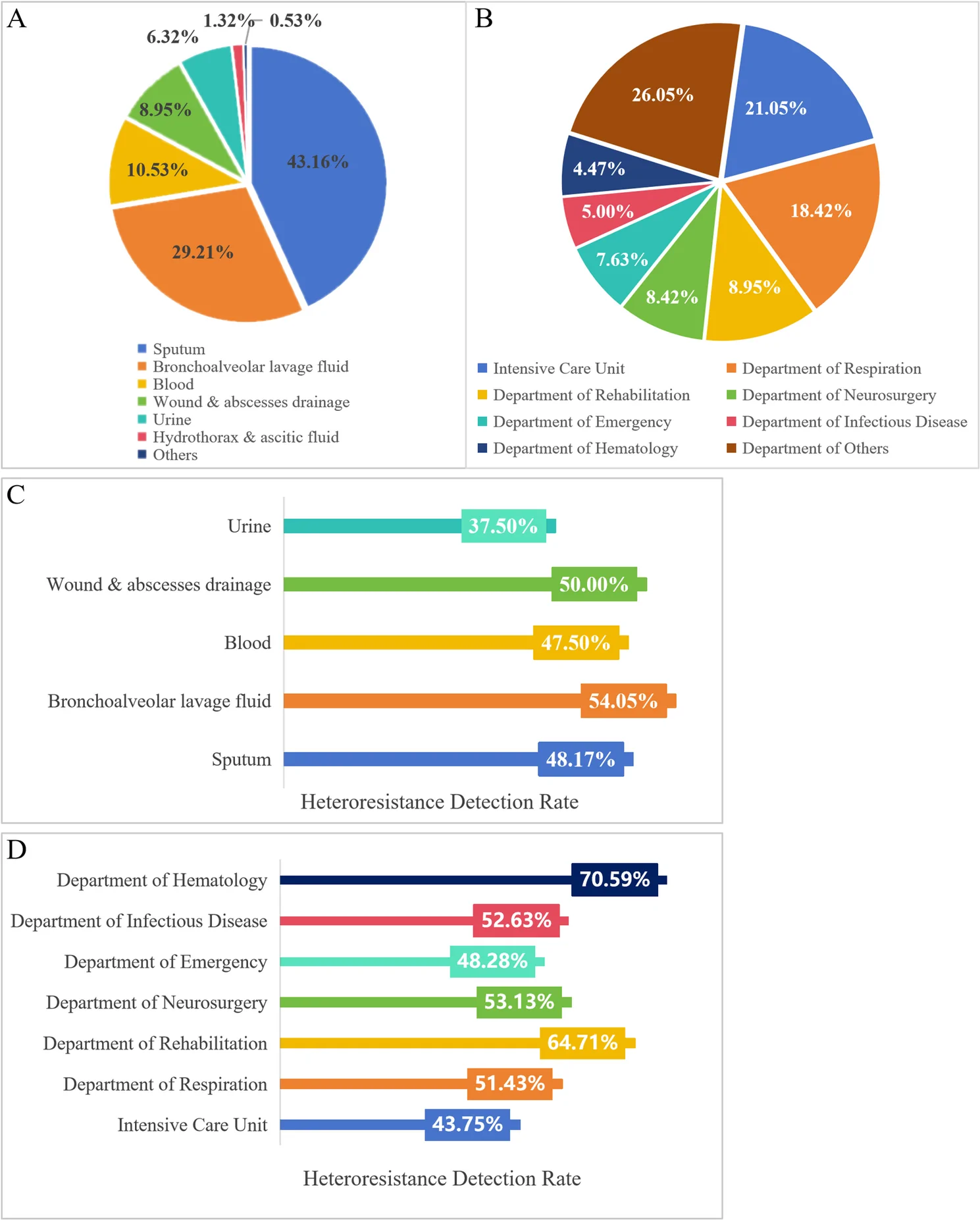
Specimen type composition, Departments distribution, and heteroresistance detection rates in *P. aeruginosa* strains. Chi-square tests were used to compare heteroresistance detection rates across different specimen types and major clinical departments. **A**. Specimen type composition of *P. aeruginosa* strains. **B**. Department distribution of *P. aeruginosa* strains. **C**. Heteroresistance detection rate among the top five specimen types. **D**. Heteroresistance detection rate of *P. aeruginosa* strains in major departments

Figure 5A illustrated the distribution of specimen types among *P. aeruginosa* strains. Among all collected isolates, sputum was the most prevalent specimen type (43.16%), followed by bronchoalveolar lavage fluid (BALF, 29.21%). Notably, these two lower respiratory tract specimens collectively accounted for over 70% of all *P. aeruginosa* isolates. Followed by blood specimens, accounting for 10.53%, with Wound & abscesses drainage (8.95%) and urine specimens (6.32%), both below 10%. This pie chart illustrates the departmental distribution of *P. aeruginosa* strains included in this study (Fig. 5B). The majority of isolates originated from the ICU (21.05%) and the respiratory department (18.42%). Meanwhile, the rehabilitation and neurosurgery departments accounted for 8.95% and 8.42%, respectively. HR detection rates in *P. aeruginosa* varied across major clinical specimen types (Fig. 5C). BALF specimens exhibited the highest rate at 54.05%, followed by wound and abscess drainage specimens at 50.00%. Meanwhile, sputum and blood specimens showed comparable rates of 48.17% and 47.50%, respectively. In contrast, urine specimens had the lowest HR detection rate at 37.50%. Despite these numerical differences, the overall chi-square test revealed no statistically significant differences among specimen types (χ² = 2.48, df = 4, *P* = 0.65). Taken together, these results indicate that, although not statistically significant, respiratory-related specimens (e.g., BALF, sputum) tend to harbour *P. aeruginosa* isolates with relatively higher HR rates. As shown in Fig. 5D, the HR detection rates of *P. aeruginosa* varied across major clinical departments. The highest rate was observed in the Hematology department (70.6%), followed by the Rehabilitation department (64.7%). Moderate rates were seen in Neurosurgery (53.1%), Infectious Disease (52.6%), and Respiratory (51.4%) departments, while the ICU exhibited the lowest rate (43.8%). Despite these numerical differences, the overall Chi-square test did not reveal statistically significant differences among departments (χ² = 6.92, df = 6, *p* = 0.328). Overall, these results indicate that although some departments had numerically higher HR rates than others, the differences were not statistically significant.

### Clonal relatedness of heteroresistant strains

**Fig. 6:**
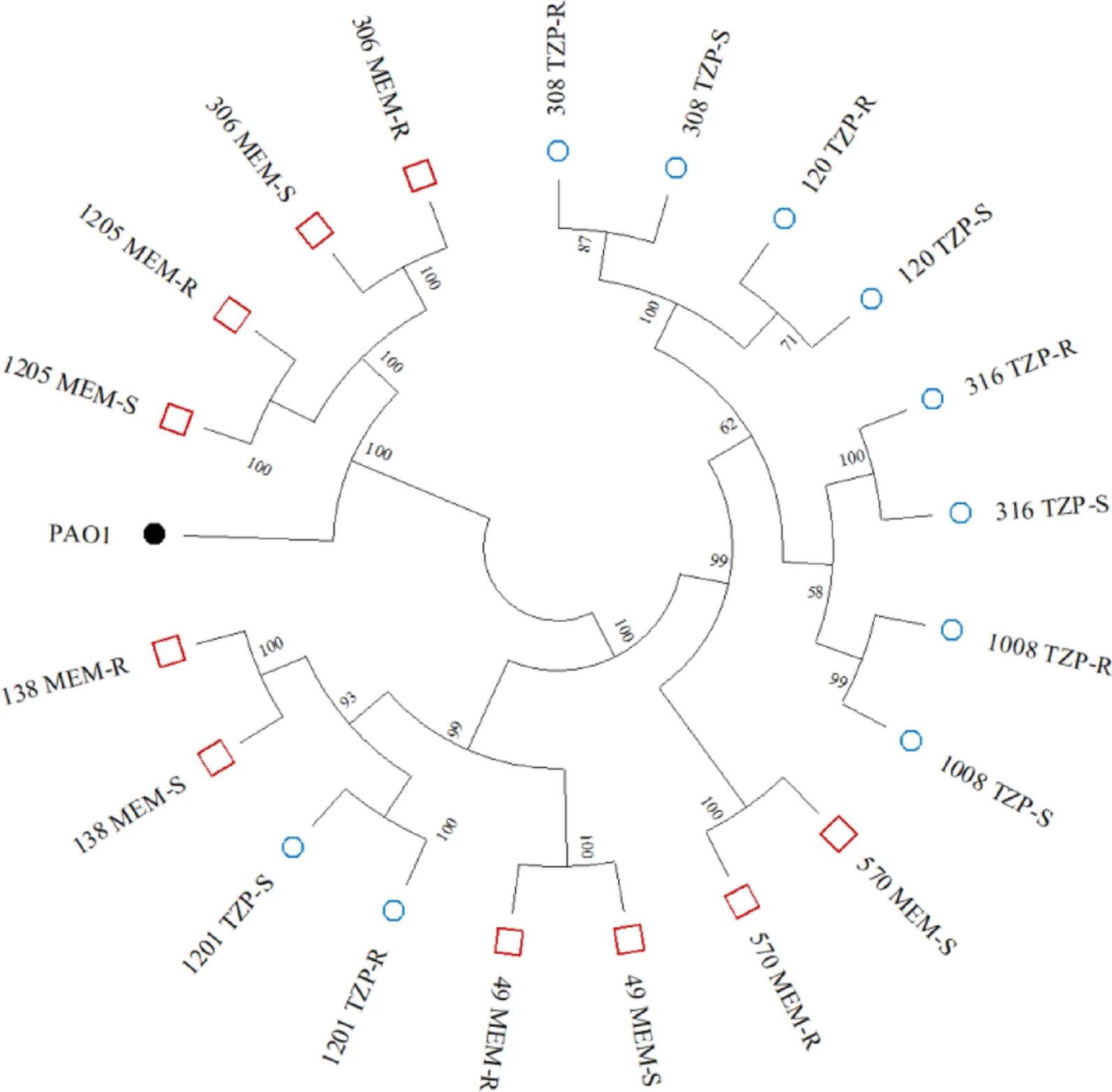
Phylogenetic tree of parental populations and their derived HR subpopulations. The tree was constructed using the Maximum Likelihood method, based on genetic distance metrics, with branch lengths representing the genetic divergence among samples. Each node is annotated with bootstrap support values, reflecting the reliability of the branching patterns. TZP-S denotes the parental strain corresponding to TZP-HR populations, while TZP-R represents the resistant subpopulation. Similarly, MEM-S (parental strain) and MEM-R (resistant subpopulation) follow this convention for MEM-HR populations

To investigate the clonal relatedness between parental populations and their derived resistant subpopulations, ten representative isolate pairs were selected to construct a phylogenetic tree based on the sequence alignment of six core resistance genes. The circular phylogenetic tree was constructed to evaluate the clonal relatedness between parental strains (denoted as “S”) and their corresponding resistant subpopulations (denoted as “R”), with PAO1 (black dot) serving as a reference strain (Fig. 6). MEM-HR and TZP-HR strains form relatively distinct clades, while the reference strain PAO1 was phylogenetically distant from all test isolates. Representative strains pairs (TZP-S/TZP-R and MEM-S/MEM-R) clustered closely within well-supported branches (bootstrap ≥ 70), demonstrating strong clonal relatedness between each parental strain and its corresponding resistant subpopulation. Overall, these findings indicated that the majority of HR strains in this study are likely monoclonal populations. Nevertheless, these findings remain preliminary and require cautious interpretation, owing to the limited sample size and restricted gene coverage.

### Clinical factors associated with heteroresistance in *P. aeruginosa* strains

To analyze the clinical correlated factors associated with HR isolates strains, we conducted univariate and multivariate analyses of various clinical and demographic variables in two patient groups: HR (*n* = 188) and non-heteroresistance (NHR, *n* = 192) (Table 2). Univariate analysis showed that, compared with patients with NHR *P. aeruginosa* infection, the HR group had a significantly lower proportion of male patients and prior antiviral exposure (both *P* < 0.05). Conversely, prior fluoroquinolone exposure was more frequent in the HR group, showing a trend toward significance (*P* < 0.1). Further multivariate logistic regression analysis confirmed that male gender (OR = 0.57, 95% CI: 0.36–0.89, *P* = 0.015) and previous antiviral exposure (OR = 0.42, 95% CI: 0.20–0.88, *P* = 0.021) were independently associated with reduced odds of HR in *P. aeruginosa*. In contrast, prior fluoroquinolone exposure (OR = 1.70; 95% CI: 1.07–2.69; *P* = 0.031) was identified as an independent risk factor, significantly increasing the likelihood of HR occurrence.

We further analyzed the correlations between the HR detection rate and corresponding clinical outcomes across different years. The temporal trends of HR detection rate exhibited inconsistent patterns with key clinical outcomes, including ICU admission rate, treatment failure rate and mortality rate (Fig. S2). In contrast, it displayed consistent trends with the duration of total length of hospital stay and postculture length of stay. Spearman rank correlation analysis, performed across 5 annual time points using aggregate population-level data, revealed a strong, statistically significant positive correlation between the HR rate and both the median total length of hospital stay (Spearman’s ρ = 0.975, *P* = 0.005) and the median postculture length of stay (Spearman’s ρ = 0.900, *P* = 0.037) (Table S3). In contrast, no statistically significant correlations were detected between the HR rate and ICU admission rate (*P* = 0.29), treatment failure rate (*P* = 0.75), or mortality rate (*P* = 0.62). Given the limited number of time points (*n* = 5), these findings should be interpreted as exploratory temporal trends rather than definitive causal associations.

**Table 2:** Univariate and Multivariate comparison of clinical characteristics in patients with heteroresistance to *P. aeruginosa*

Variable			Univariate		Multivariate	
	HR (*n* = 188)	NHR (*n* = 192)	OR (95% CI)	*P*-value	OR (95% CI)	*P*-value
Male gender, n (%)	119 (63.30%)	143 (74.48%)	0.59 (0.38 ~ 0.92)	**0.019**	0.57 (0.36 ~ 0.89)	**0.015**
Average Age, median (IQR)	69.5 (55.3–80)	71 (59–82)	NA	0.504		
Admission to ICU, n (%)	57 (30.32%)	66 (34.38%)	0.83 (0.54 ~ 1.28)	0.398		
Chronic conditions on admission						
Hypertension	106 (56.38%)	102 (53.13%)	1.14 (0.76 ~ 1.71)	0.524		
Diabetes	66 (35.11%)	61 (31.77%)	1.16 (0.76 ~ 1.78)	0.491		
Solid malignancy	27 (14.36%)	32 (16.67%)	0.84 (0.48 ~ 1.46)	0.535		
Chronic kidney disease	37 (19.68%)	42 (21.88%)	0.88 (0.53 ~ 1.44)	0.598		
Cardiovascular disease	69 (36.70%)	69 (35.94%)	1.03 (0.68 ~ 1.57)	0.877		
Cerebrovascular disease	81 (43.09%)	97 (50.52%)	0.74 (0.49 ~ 1.11)	0.147		
Haematological Diseases	14 (7.45%)	12 (6.25%)	1.21 (0.54 ~ 2.68)	0.644		
Respiratory disease	52 (27.66%)	64 (33.33%)	0.76 (0.49 ~ 1.19)	0.23		
Hypoproteinemia	74 (39.36%)	74 (38.54%)	1.04 (0.69 ~ 1.56)	0.87		
Corticosteroids within 4 weeks	21 (11.17%)	18 (9.38%)	1.22 (0.63 ~ 2.36)	0.565		
Bedridden	49 (26.06%)	41 (21.35%)	1.30 (0.81 ~ 2.09)	0.281		
Multiple hospitalizations history	42 (22.34%)	49 (25.52%)	0.84 (0.52 ~ 1.35)	0.468		
Infection type						
Pulmonary Infection	141 (75.00%)	146 (76.04%)	0.95 (0.59 ~ 1.51)	0.813		
Bacteraemia	46 (24.47%)	52 (27.08%)	0.85 (0.52 ~ 1.39)	0.522		
Other infections	56 (29.79%)	66 (34.38%)	0.81 (0.53 ~ 1.25)	0.339		
Invasive procedures within prior 4 weeks						
Central venous catheter	55 (29.26%)	62 (32.29%)	0.87 (0.56 ~ 1.34)	0.522		
Arteriosus catheter	36 (19.15%)	41 (21.35%)	0.87 (0.53 ~ 1.44)	0.593		
Mechanical ventilation	70 (37.23%)	66 (34.38%)	1.13 (0.74 ~ 1.72)	0.561		
Drainage tube	70 (37.23%)	68 (35.42%)	1.08 (0.71 ~ 1.64)	0.713		
Parenteral nutrition	11 (5.85%)	13 (6.77%)	0.86 (0.37 ~ 1.96)	0.713		
Surgery in the past 6 months	39 (20.74%)	39 (20.31%)	1.03 (0.62 ~ 1.69)	0.917		
Antimicrobial exposure within 1 months						
Cephalosporins	138 (73.40%)	133 (69.27%)	1.27 (0.85 ~ 1.90)	0.373		
Carbapenems	62 (32.98%)	57 (29.69%)	1.17 (0.76 ~ 1.80)	0.489		
Fluoroquinolone	64 (34.04%)	50 (26.04%)	1.47 (0.94 ~ 2.28)	**0.09**	1.70 (1.07 ~ 2.69)	**0.031**
Aminoglycosides	22 (11.70%)	27 (14.06%)	0.81 (0.44 ~ 1.48)	0.493		
Tigecycline	10 (5.32%)	11 (5.73%)	0.92 (0.38 ~ 2.23)	0.861		
Antifungal drugs	38 (20.21%)	45 (23.44%)	0.83 (0.51 ~ 1.35)	0.447		
Antiviral drugs	12 (6.38%)	24 (12.50%)	0.48 (0.23 ~ 0.98)	**0.045**	0.42 (0.20 ~ 0.88)	**0.021**

HR, heteroresistant group (*n* = 188); NHR, non-heteroresistant group (*n* = 192). OR, odds ratio; CI, confidence interval. All categorical variables were coded as 1 (“positive/present”) or 0 (“negative/absent”), with the “negative/absent” category as the reference. Only counts and percentages for the “positive/present” category are shown in the table. An OR > 1 indicates increased odds of heteroresistance, whereas an OR < 1 indicates decreased odds

## Discussion

This study provides a comprehensive foundation for understanding the epidemiology of HR in clinical *P. aeruginosa* strains by characterizing the longitudinal temporal trends and phenotypic profiles of HR to TZP and MEM over a 14-year period (2011–2024).

Our results indicate a high prevalence of HR among clinically isolated *P. aeruginosa* strains, with a confirmed HR rate of 49.5% to TZP or MEM. Notably, PAP was performed on all 380 isolates, rather than only those identified as potential heteroresistant isolates in preliminary screening. This approach allowed the detection of low-frequency resistant subpopulations that might have been missed by initial screening methods, providing a more comprehensive assessment of HR prevalence. The prevalence of HR to different antibiotics exhibited significant variability, primarily associated with the strain selection, the detection methods and criteria used to determine HR [4]. In Chen’s research using the PAPs assay found that the HR rate to MEM was as high as 58.2%, and 32.4% showed a cross-HR to all the four antibiotics, provided valuable insights into the complex resistance patterns within *P. aeruginosa* populations [15]. Other studies have revealed that invasive *P. aeruginosa* strains exhibited HR rates of 84.9% to carbapenems and 57.3% to FEP [14, 18]. In contrast, the prevalence of HR to TZP among *Klebsiella pneumoniae* bloodstream infection (BSI) isolates was 6%, lower than our findings [16]. A notable and unanticipated finding was that 9.5% of strains displayed dual-antibiotic HR, which may pose a significant threat to antimicrobial therapy, potentially complicating treatment strategies and contributing to treatment failure. Distinct heteroresistant subpopulations can further drive genotype diversification in the remaining population by secreting signaling molecules or metabolites through quorum sensing systems, enabling less resistant cells to acquire higher resistance to additional antibiotics [19, 20]. Therefore, it is crucial to determine the prevalence of HR in *P. aeruginosa* isolates in the local area to optimize the selection of initial empirical antibiotic treatments and improve clinical decision-making.

Our results show that the HR rates for both MEM and TZP exhibited a consistent pattern of first increasing and then decreasing, peaking in 2017–2019 before starting to decline. This trend may be attributed to the implementation and impact of the National Action Plan for Containing Bacterial Resistance in 2016, which aims to further standardize antibiotic use across various sectors [21–23]. Monitoring in a tertiary hospital in southern China from 2014 to 2018 revealed a year-on-year decrease in antimicrobial use, which correlated with a marked decline in the resistance trend of *P. aeruginosa* [24]. In contrast, our research observed an upward trend in HR. A study conducted from 2011 to 2015 indicated that there was a slight increase in carbapenem HR among *P. aeruginosa* strains, which was positively correlated with carbapenem consumption [13]. In our study, MEM-HR increased from 2011 to 2019, followed by a subsequent decline. Interestingly, the HR rate was the lowest during the 2020–2022 pandemic, likely due to the stringent infection prevention and control protocols in healthcare settings during that period. A recent study showed that the incidence of multidrug-resistant (MDR) and extensively drug-resistant (XDR) *P. aeruginosa* infections increased among ICU patients during the pandemic, possibly due to the widespread use of broad-spectrum antibiotics increasing resistance pressure [25, 26]. The increase in drug-resistant strains can also lead to a temporary decrease in the HR detection rates. Piperacillin/tazobactam, a broad- spectrum β-lactam/β-lactamase inhibitor combination, is commonly used as empirical or targeted therapy for hospitalized patients. As a result, the rate of TZP-HR has remained relatively high, although it has shown a slight decline in recent years. Nevertheless, it remains higher than the HR rate observed in *Klebsiella pneumoniae* [16]. Therefore, continuous surveillance of the prevalence of HR in local areas is necessary to inform more rational antibiotic selection in clinical practice. Additionally, our unique 14-year longitudinal analysis uniquely captures the impact of national policy interventions and pandemic-era infection control measures on HR dynamics.

Our research findings indicated that the resistance phenotypes of the majority of HR subpopulations (approximately 81%) increased, including the S - R, S - I, and I - R profiles. Moreover, the frequencies of these resistant subpopulations ranged from 10⁻⁷ to 2 × 10⁻⁴. Previous studies have demonstrated that exposure to β-lactams and carbapenems can selectively enrich pre-existing heteroresistant subpopulations in *P. aeruginosa*, with reported frequencies typically ranging from 10⁻⁷ to 10⁻⁴ [4, 27, 28]. Our observed HR subpopulation frequencies for both TZP and MEM (10⁻⁷–2 × 10⁻⁴) are consistent with these findings, supporting the notion that antibiotic selective pressure plays a critical role in shaping HR dynamics rather than inducing de novo resistance. In Andersson’s research, they also identified similar subpopulations that play a crucial role in altering antibiotic susceptibility patterns. Although these subpopulations generally do not affect the resistance of the overall bacterial population due to their low frequencies [29]. A recent study by David S Weiss showed that HR as the primary driver of inconsistencies in antibiotic susceptibility testing (AST), particularly within heteroresistant subpopulations characterized by moderate frequencies of resistant cells, which account for nearly all discrepancies with standard AST [30]. Therefore, clarifying the occurrence frequencies of heteroresistant subpopulations is of great significance for understanding the complex nature of bacterial resistance and for more targeted antimicrobial strategies, while also potentially guiding future recommendations and selection of antibiotic breakpoints.

Through univariate and multivariate logistic regression analyses, we found that male gender and prior antiviral exposure were independently associated with reduced odds of HR in *P. aeruginosa*. This finding contrasts with previous reports suggesting that male sex was an independent risk factors for the acquisition of imipenem-heteroresistant isolates [14]. The underlying reasons for this discrepancy remain unclear and warrant further investigation. Exposure to antiviral drugs may also influence the development of HR. In vitro exposure of bacteria to antiviral agents can induce multidrug-resistant mutations, potentially driving cross-resistance to antibiotics. In Escherichia coli strains exposed to antiviral drugs, there are numerous unique base mutations, insertions, and deletions in genes associated with antibacterial resistance. These genetic changes may influence the expression of resistance - related proteins and modify the bacterial resistance phenotypes [31]. Interestingly, our findings demonstrated that prior fluoroquinolone exposure was an independent risk factor for HR, consistent with the findings of Yun Xia. Yun Xia et al. reported that previous fluoroquinolone treatment was identified as an independent risk factor for the acquisition tigecycline HR (TG-HR) in *E. cloacae* [32]. This could be explained by the shared efflux system (MexAB and MexCD) between fluoroquinolones and MEM in *P. aeruginosa*, which promote the upregulation of RND efflux pumps, enhancing may directly contribute to bacterial resistance [33]. However, this study lacks detailed information on prior antiviral and antibiotic exposures, including drug type, indication, dosage, duration, and timing of administration. Given that the development of HR may be influenced by the intensity and duration of antimicrobial exposure, this limitation should be considered when interpreting the regression analyses. Future studies incorporating more comprehensive exposure data are warranted to better elucidate the relationship between clinical antiviral and antibiotic use and the emergence of HR in *P. aeruginosa*. At the group level, our results indicated that HR was not significantly associated with treatment failure, in contrast to some previous reports [8, 34]. This discrepancy may reflect differences in patient populations, antimicrobial regimens, or the definitions of treatment failure used across studies. Conversely, HR may be associated with longer hospital stays, although this conclusion remains controversial [14, 18]. Collectively, these findings indicate that while HR is prevalent, its clinical impact may vary depending on patient characteristics, antimicrobial exposure, and study context.

This study has several inherent limitations. Primarily, the retrospective study was conducted within a single tertiary hospital, which may limit the generalizability of the findings to other multicenter settings. Secondly, we only used the PAP method for TZP and MEM, and the HR to other commonly used anti - Pseudomonas agents remain to be further verified. Additionally, clonal analysis in this study was conducted on a limited number of representative isolates and targeted only six common resistance-associated genes. Although our results indicate close genetic relatedness between parental strains and HR subpopulations, these findings may not be broadly generalizable. Future investigations with larger sample cohorts and comprehensive whole-genome sequencing are required to better characterize the clonal relationships of HR strains in *P. aeruginosa*. As HR continues to evolve and become more complex, future research should focus on exploring more effective detection methods, prevention strategies, and antibiotic treatment approaches to tackle this multifaceted challenge.

## Conclusion

This study highlights the widespread prevalence, complex temporal dynamics, and diverse phenotypic characteristics of HR in *P. aeruginosa*, revealing significant fluctuations in HR rates over time. Our findings underscore the critical role of factors such as gender, prior exposure to antiviral agents and fluoroquinolone antibiotics in the development of HR. Notably, HR was not significantly associated with treatment failure in this study, although this finding contrasts with some previous reports—likely reflecting variations in patient populations, antimicrobial regimens, or definitions of treatment failure across studies. Interestingly, HR detection rates for both MEM and TZP exhibited distinct peaks during 2017–2019, followed by a decline during the 2020–2022 pandemic period. These results underscore the potential clinical value of integrating HR screening into antimicrobial susceptibility testing, particularly for high-risk populations, to better guide therapy and mitigate the risk of treatment failure.

## Supplementary Information

Below is the link to the electronic supplementary material.

Supplementary Material 1.

## Data Availability

No datasets were generated or analysed during the current study.

## Abbreviations

HR: Heteroresistance
TZP: Piperacillin/Tazobactam
MEM: Meropenem
PAP: Population analysis profile assay
K-B: Kirby-Bauer disk diffusion
Etest: Etest gradient diffusion assay
MIC: Minimum inhibitory concentration
ICU: Intensive care unit
FEP: Cefepime
CAZ: Ceftazidime
LEV: Levofloxacin
AK: Amikacin
IPM: Imipenem
TG: Tigecycline
S-S: Susceptible to reduced susceptibility
S-I: Susceptible to intermediate susceptibility
I-R: Intermediate to resistant transition
S-R: Susceptible to resistant
R-R: Resistance to higher-level resistance
BALF: Bronchoalveolar lavage fluid
NHR: Non-heteroresistance
MDR: Multidrug-resistant
XDR: Extensively drug-resistant
AST: Antibiotic susceptibility testing

## References

[1] Lister PD, Wolter DJ, Hanson ND. Antibacterial-resistant Pseudomonas aeruginosa: clinical impact and complex regulation of chromosomally encoded resistance mechanisms. Clin Microbiol Rev. 2009;22:582–610. 10.1128/CMR.00040-09. 19822890 PMC2772362

[2] Qin S, Xiao W, Zhou C, Pu Q, Deng X, Lan L, et al. Pseudomonas aeruginosa: pathogenesis, virulence factors, antibiotic resistance, interaction with host, technology advances and emerging therapeutics. Signal Transduct Target Ther. 2022;7:199. 10.1038/s41392-022-01056-1. 35752612 PMC9233671

[3] Abbott CN, Dhillon A, Timalsina S, Furr E, Velicitat P, Belley A et al. The association between undetected heteroresistance and antibiotic treatment failure in complicated urinary tract infection. medRxiv. 2025.03.11.25323422. 10.1101/2025.03.11.25323422

[4] El-Halfawy OM, Valvano MA. Antimicrobial Heteroresistance: an Emerging Field in Need of Clarity. Clin Microbiol Rev. 2015;28:191–207. 10.1128/CMR.00058-14. 25567227 PMC4284305

[5] Roch M, Sierra R, Andrey DO. Antibiotic heteroresistance in ESKAPE pathogens, from bench to bedside. Clin Microbiol Infect. 2022;S1198. 10.1016/j.cmi.2022.10.018. -743X(22)00532-8. 36270588

[6] Benquan W, Yingchun T, Kouxing Z, Tiantuo Z, Jiaxing Z, Shuqing T. Staphylococcus heterogeneously resistant to vancomycin in China and antimicrobial activities of imipenem and vancomycin in combination against it. J Clin Microbiol. 2002;40:1109–12. 10.1128/JCM.40.3.1109-1112.2002. 11880455 PMC120272

[7] Chaïbi K, Nagle S, Billard-Pomares T, Zahar JR, Roux D. Heteroresistance in Pseudomonas aeruginosa ventilator-associated pneumonia: a neglected hypothesis for a clinical impasse. Clin Microbiol Infect. 2026;32:210–3. 10.1016/j.cmi.2025.10.018. 41173338

[8] Band VI, Weiss DS, Heteroresistance. A cause of unexplained antibiotic treatment failure? PLoS Pathog. 2019;15:e1007726. 10.1371/journal.ppat.1007726. 31170271 PMC6553791

[9] Alexander HE. Mode of action of streptomycin on type B Hemophilus influenzae. Am J Dis Child (1911). 1948;75:428–30. 18882067

[10] Lin S-Y, Chen T-C, Chen F-J, Chen Y-H, Lin Y-I, Kristopher Siu L, et al. Molecular epidemiology and clinical characteristics of hetero-resistant vancomycin intermediate Staphylococcus aureus bacteremia in a Taiwan Medical Center. J Microbiol Immunol Infect. 2012;45:435–41. 10.1016/j.jmii.2012.05.004. 22749725

[11] Li J, Rayner CR, Nation RL, Owen RJ, Spelman D, Tan KE, et al. Heteroresistance to colistin in multidrug-resistant Acinetobacter baumannii. Antimicrob Agents Chemother. 2006;50:2946–50. 10.1128/AAC.00103-06. 16940086 PMC1563544

[12] Lin JY, Zhu ZC, Zhu J, Chen L, Du H. Antibiotic heteroresistance in Klebsiella pneumoniae: Definition, detection methods, mechanisms, and combination therapy. Microbiol Res. 2024;283:127701. 10.1016/j.micres.2024.127701. 38518451

[13] Rodríguez-Villodres Á, de la Ortiz JM, Álvarez-Marín R, Pachón J, Aznar J, Lepe JA, et al. Heteroresistance to Piperacillin-Tazobactam in Clinical Isolates of Escherichia coli Sequence Type 131. Antimicrob Agents Chemother. 2017;62:e01923–17. 10.1128/AAC.01923-17. 29061749 PMC5740306

[14] He J, Jia X, Yang S, Xu X, Sun K, Li C, et al. Heteroresistance to carbapenems in invasive Pseudomonas aeruginosa infections. Int J Antimicrob Agents. 2018;51:413–21. 10.1016/j.ijantimicag.2017.10.014. 29127047

[15] Lu Y, Liu Y, Zhou C, Liu Y, Long Y, Lin D, et al. Quorum sensing regulates heteroresistance in Pseudomonas aeruginosa. Front Microbiol. 2022;13:1017707. 10.3389/fmicb.2022.1017707. 36386621 PMC9650436

[16] Babiker A, Lohsen S, Van Riel J, Hjort K, Weiss DS, Andersson DI, et al. Heteroresistance to piperacillin/tazobactam in Klebsiella pneumoniae is mediated by increased copy number of multiple β-lactamase genes. JAC Antimicrob Resist [Internet]. 2024;6:dlae057. 10.1093/jacamr/dlae057. 38601791 PMC11004786

[17] Nicoloff H, Hjort K, Levin BR, Andersson DI. The high prevalence of antibiotic heteroresistance in pathogenic bacteria is mainly caused by gene amplification. Nat Microbiol. 2019;4:504–14. 10.1038/s41564-018-0342-0. 30742072

[18] Jia X, Ma W, He J, Tian X, Liu H, Zou H, et al. Heteroresistance to cefepime in Pseudomonas aeruginosa bacteraemia. Int J Antimicrob Agents. 2020;55:105832. 10.1016/j.ijantimicag.2019.10.013. 31669739

[19] Lee HH, Molla MN, Cantor CR, Collins JJ. Bacterial charity work leads to population-wide resistance. Nat Publishing Group. 2010;467:82–5. 10.1038/nature09354. PMC2936489 20811456

[20] Vallet-Gely I, Opota O, Boniface A, Novikov A, Lemaitre B. A secondary metabolite acting as a signalling molecule controls Pseudomonas entomophila virulence. Cell Microbiol. 2010;12:1666–79. 10.1111/j.1462-5822.2010.01501.x. 20597908

[21] Han haochen. National Action Plan for Containing Bacterial Drug Resistance in 2016. https://www.gov.cn/xinwen/2016-08/25/content_5102348.htm. Accessed 5 Dec 2024.

[22] Recio-Rufián M, López-Viñau T, Gálvez-Soto V, Cano Á, Ruiz-Montero R, Gutiérrez-Gutiérrez B, et al. Incidence, clinical and genomic trends of hospital- and Non-hospital-onset KPC-producing Klebsiella pneumoniae infections before and during the COVID-19 era: a ten-year interrupted time series study. Antimicrob Resist Infect Control. 2025;14:97. 10.1186/s13756-025-01614-6. 40775366 PMC12333191

[23] Ding G, Chen Y, Shi C, Vinturache A, Zhang Y. Trends in antimicrobial resistance of bacterial isolates in China, 2014 to 2022. Clin Microbiol Infect. 2024;30:1475–7. 10.1016/j.cmi.2024.07.011. 39032554

[24] Zeng S, Xu Z, Wang X, Liu W, Qian L, Chen X, et al. Time series analysis of antibacterial usage and bacterial resistance in China: observations from a tertiary hospital from 2014 to 2018. Infect Drug Resist. 2019;12:2683–91. 10.2147/IDR.S220183. 31695444 PMC6717838

[25] Serretiello E, Manente R, Dell’Annunziata F, Folliero V, Iervolino D, Casolaro V, et al. Antimicrobial Resistance in Pseudomonas aeruginosa before and during the COVID-19 Pandemic. Microorganisms. 2023;11:1918. 10.3390/microorganisms11081918. 37630478 PMC10458743

[26] Gysin M, Acevedo CT, Haldimann K, Bodendoerfer E, Imkamp F, Bulut K, et al. Antimicrobial susceptibility patterns of respiratory Gram-negative bacterial isolates from COVID-19 patients in Switzerland. Ann Clin Microbiol Antimicrob. 2021;20:64. 10.1186/s12941-021-00468-1. 34493302 PMC8422836

[27] Oikonomou O, Panopoulou M, Ikonomidis A. Investigation of carbapenem heteroresistance among different sequence types of Pseudomonas aeruginosa clinical isolates reveals further diversity. J Med Microbiol [Internet] Microbiol Soc. 2011;60:1556–8. 10.1099/jmm.0.032276-0. 21596913

[28] Lin J, Xu C, Fang R, Cao J, Zhang X, Zhao Y, et al. Resistance and Heteroresistance to Colistin in Pseudomonas aeruginosa Isolates from Wenzhou, China. Antimicrob Agents Chemother. 2019;63:e00556–19. 10.1128/AAC.00556-19. 31383654 PMC6761505

[29] Andersson DI, Nicoloff H, Hjort K. Mechanisms and clinical relevance of bacterial heteroresistance. Nat Rev Microbiol. 2019;17:479–96. 10.1038/s41579-019-0218-1. 31235888

[30] Ozturk T, Weiss DS. Heteroresistance is a cause of discrepant antibiotic susceptibility testing results. Lancet Microbe Elsevier. 2024;5:e312. 10.1016/S2666-5247(23)00374-9 38244556

[31] Wallace VJ, Sakowski EG, Preheim SP, Prasse C. Bacteria exposed to antiviral drugs develop antibiotic cross-resistance and unique resistance profiles. Commun Biol. 2023;6:837. 10.1038/s42003-023-05177-3. 37573457 PMC10423222

[32] Liu H, Jia X, Zou H, Sun S, Li S, Wang Y, et al. Detection and characterization of tigecycline heteroresistance in E. cloacae: clinical and microbiological findings. Emerg Microbes Infect. 2019;8:564–74. 10.1080/22221751.2019.1601031. 30945610 PMC6455127

[33] Quale J, Bratu S, Gupta J, Landman D. Interplay of efflux system, ampC, and oprD expression in carbapenem resistance of Pseudomonas aeruginosa clinical isolates. Antimicrob Agents Chemother. 2006;50:1633–41. 10.1128/AAC.50.5.1633-1641.2006. 16641429 PMC1472219

[34] Band VI, Satola SW, Burd EM, Farley MM, Jacob JT, Weiss DS. Carbapenem-Resistant Klebsiella pneumoniae Exhibiting Clinically Undetected Colistin Heteroresistance Leads to Treatment Failure in a Murine Model of Infection. mBio. 2018;9:e02448–17. 10.1128/mBio.02448-17. 29511071 PMC5844991

