# Supplementary material for: Clinical characteristics and temporal trends of meropenem and piperacillin/tazobactam heteroresistance in Pseudomonas aeruginosa isolates
Source: Ann Clin Microbiol Antimicrob. 2026 Jun 26;25:36. doi: 10.1186/s12941-026-00875-2 (PMC13584257; doi:10.1186/s12941-026-00875-2)
Supplement: Supplementary file 1 [file 12941_2026_875_MOESM1_ESM.docx]

Log10 cfu/ml

| TZP:ug/ml | ATCC27853(S） | HR1 | HR2 | HR3 | HR4 | R |
| --- | --- | --- | --- | --- | --- | --- |
| 0.5 | 8 | 8 | 8 | 8 | 8 | 8 |
| 1 | 8 | 8 | 8 | 8 | 8 | 8 |
| 2 | 2.7 | 5 | 8 | 8 | 8 | 8 |
| 4 | 1 | 2.2 | 8 | 8 | 8 | 8 |
| 8 | 0 | 2 | 4 | 5 | 7 | 8 |
| 16 | 0 | 1.8 | 4 | 2 | 7 | 8 |
| 32 | 0 | 0.6 | 2 | 0.6 | 6 | 8 |
| 64 | 0 | 0.7 | 0.7 | 0 | 3 | 8 |
| 128 | 0 | 0 | 0 | 0 | 1.6 | 5 |
| 256 | 0 | 0 | 0 | 0 | 0 | 0 |

Log10 cfu/ml

| MEM:ug/ml | ATCC27853(S) | HR1 | HR2 | HR3 | HR4 | R |
| --- | --- | --- | --- | --- | --- | --- |
| 0 | 8 | 8 | 8 | 8 | 8 | 8 |
| 0.25 | 7 | 8 | 7 | 8 | 5 | 8 |
| 0.5 | 2.07918 | 6 | 6 | 7 | 4 | 8 |
| 1 | 1.27875 | 6 | 4 | 7 | 2.30103 | 8 |
| 2 | 0 | 4 | 2.30103 | 6 | 1.74819 | 7.6 |
| 4 | 0 | 4 | 1.99123 | 5 | 1.62325 | 7.4 |
| 8 | 0 | 3 | 1.41497 | 4 | 1.60206 | 3 |
| 16 | 0 | 2 | 0 | 2.69897 | 0 | 0 |
| 32 | 0 | 0 | 0 | 0.778151 | 0 | 0 |

**Fig. S1**. Population analysis profiles (PAP) of the Piperacillin-tazobactam heteroresistant (TZP-HR) and meropenem-heteroresistant (MEM-HR) isolates and the control strain (P. aeruginosa ATCC 27853, R-resistant)

|  | Total length of hospital stay (median, days) | Postculture length of stay(median, days) | Heteroresistant rate | ICU admission rate | Treatment failure rate | Mortality rate |
| --- | --- | --- | --- | --- | --- | --- |
| 2011-2013 | 16 | 7 | 34.2857% | 14.2857% | 62.8571% | 11.4286% |
| 2014-2016 | 25.5 | 15 | 47.8261% | 43.4783% | 84.7826% | 10.8696% |
| 2017-2019 | 29 | 16 | 64.7059% | 37.2549% | 72.549% | 7.84314% |
| 2020-2022 | 25.5 | 14.5 | 54.8077% | 38.4615% | 66.3462% | 12.5% |
| 2023-2024 | 24 | 14 | 45.8333% | 27.0833% | 75.6944% | 9.72222% |

**Fig. S2**. Temporal trends in heteroresistance rates and key clinical outcomes (ICU admission, treatment failure, mortality and median length of hospital stay) among *P. aeruginosa* isolates, 2011-2024

**Table S1**. PCR amplification primers used in this study

| **Primers** | **Sequence (5′–3′)** |
| --- | --- |
| AmpC-F | GGGCTGGCCTCGAAAGAGGAC |
| AmpC-R | CATGTCGCCGACCTTGTAGTAACC |
| mexB-F | TCGTGTTCCTGGTGATGTACC |
| mexB-R | GACACGATGGTGATGGAGAACT |
| oprD-F | CGCCGACAAGAAGAACTAGC |
| oprD-R | GTCGATTACAGGATCGACAG |
| ftsL-F | GATCACGCAGAACCGCCAT |
| ftsL-R | AGAAGAACTCAAGGCCAACCC |
| gyrA-F | ATCTCGACTACGCGATGAGC |
| gyrA-R | GATACCGCTGGAACCGTTGA |
| Aph-F | CATGATGCAGCCACCTCCA |
| Aph-R | TCCAATAGCCTGAAGTACGCC |

**Table S2.** Gender and Age Characteristics of heteroresistance in *P. aeruginosa* strains

| age | PAE | HR | HR proportion | HR detection rate | P-value |
| --- | --- | --- | --- | --- | --- |
| ≤60 | 112 | 61 | 32.45% | 54.46% | P>0.05 |
| 60-80 | 160 | 79 | 42.02% | 49.38% |  |
| ≥80 | 108 | 48 | 25.53% | 44.44% |  |
| gender |  |  |  |  |  |
| Male | 262 | 119 | 63.30% | 45.42% | P<0.05 |
| Female | 118 | 69 | 36.70% | 58.47% |  |

**Table S3.** Spearman rank correlation analysis between *P. aeruginosa* heteroresistance rates and key clinical outcomes across five annual time points

| **Clinical Outcome Variable** | **Spearman’s ρ** | ***P* - value** | **95% Confidence Interval** |
| --- | --- | --- | --- |
| ICU admission rate | 0.6 | 0.285 | -1.00-1.00 |
| Treatment failure  rate | 0.2 | 0.747 | -1.00-1.00 |
| Mortality rate | -0.31 | 0.624 | -1.00-1.00 |
| Total length of hospital stay (days), median | 0.975 | 0.005* | 0.745-1.00 |
| Postculture length of  stay (days), median | 0.9 | 0.037* | 0.111-1 |

Note: P<0.05 was considered statistically significant denoted by *; Association strength was defined as: weak (∣ρ∣<0.4), moderate (0.4≤∣ρ∣<0.7), strong (∣ρ∣≥0.7).
